# Correction to: Lagrangian mechanics of active systems

**DOI:** 10.1140/epje/s10189-022-00170-w

**Published:** 2022-03-07

**Authors:** Anton Solovev, Benjamin M. Friedrich

**Affiliations:** TU Dresden, Dresden, Germany

## Correction to: Eur Phys J E 10.1140/epje/s10189-021-00016-x

In Fig. 3D, the hydrodynamic interaction $\Gamma _{12}$ corresponds to a direction angle $\psi =5\pi /6$ (instead of $\psi =2\pi /3$, as incorrectly written in the figure caption). This does not change any of the conclusion of the paper.

The original article has been corrected.

