# Supplementary figures and images for: Lagrangian mechanics of active systems
Source: Eur Phys J E Soft Matter. 2021 Apr 8;44(4):49. doi: 10.1140/epje/s10189-021-00016-x (PMC8032648; doi:10.1140/epje/s10189-021-00016-x)

**A** Triangulated mesh for cilia pair

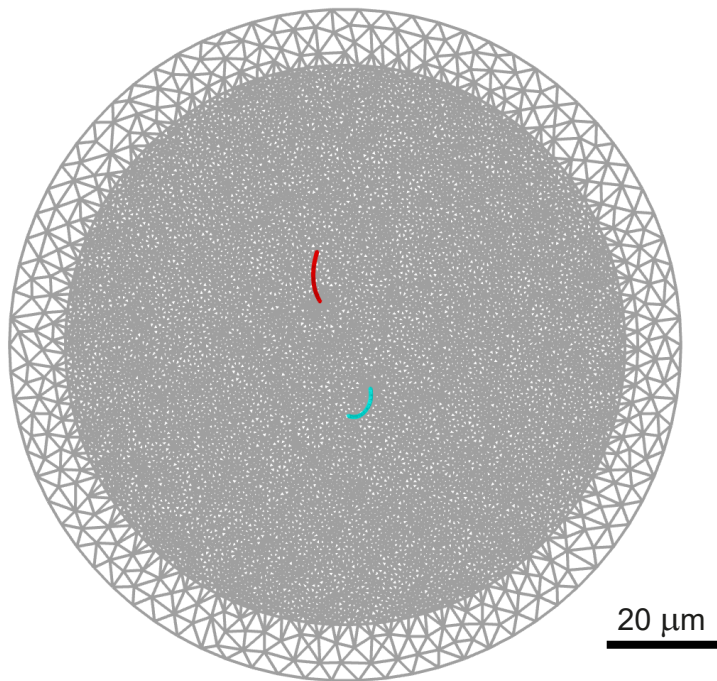

**B** Close-up

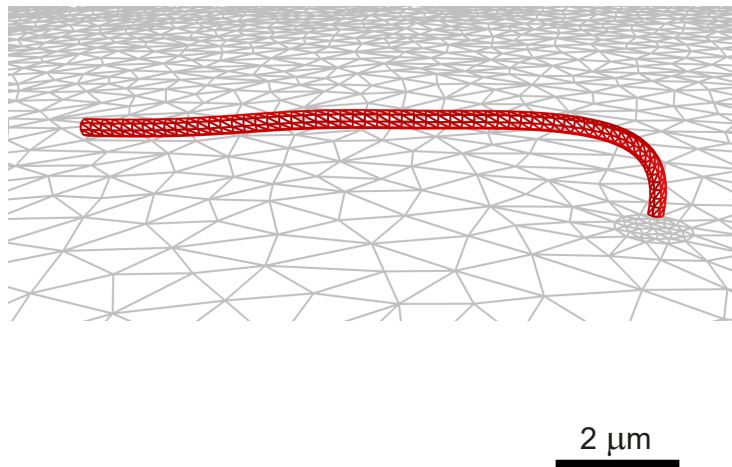

Supplement: Supplementary file 1 — (pdf 890 KB) [file 10189_2021_16_MOESM1_ESM.pdf]

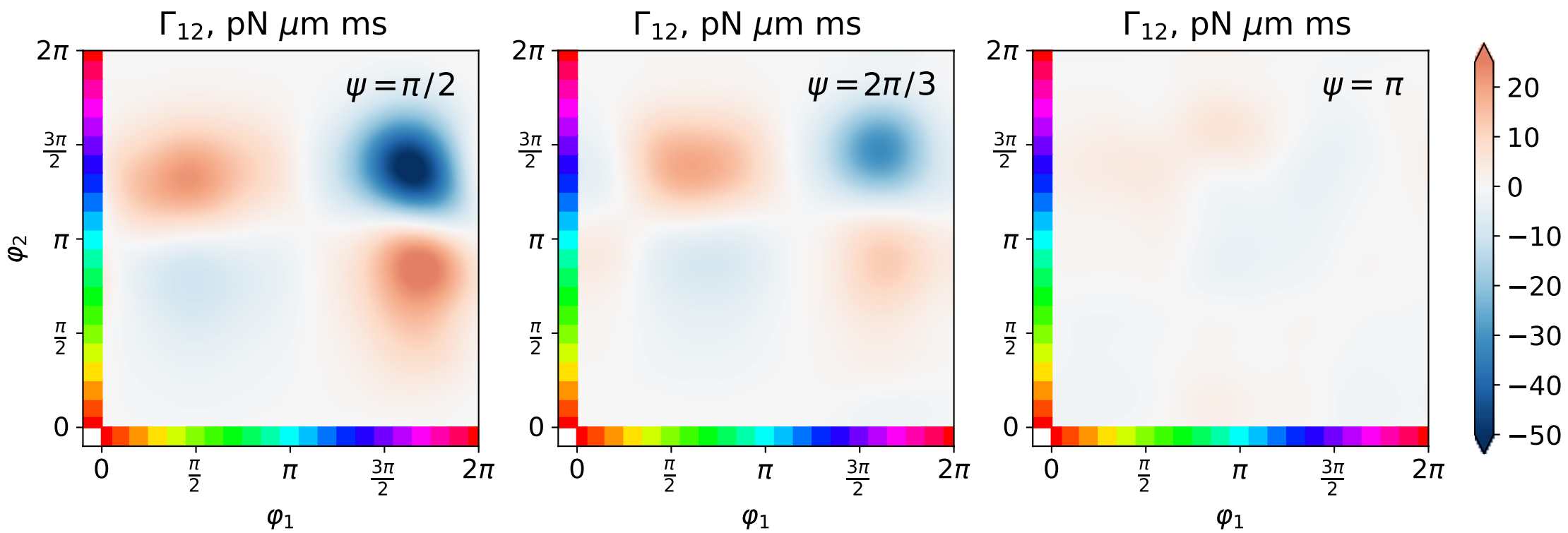

Supplement: Supplementary file 2 — (pdf 432 KB) [file 10189_2021_16_MOESM2_ESM.pdf]
